# Dynamical Consequences of Bandpass Feedback Loops in a Bacterial Phosphorelay

**DOI:** 10.1371/journal.pone.0025102

**Published:** 2011-09-29

**Authors:** Shaunak Sen, Jordi Garcia-Ojalvo, Michael B. Elowitz

**Affiliations:** 1 Department of Control and Dynamical Systems, Division of Engineering and Applied Science, California Institute of Technology, Pasadena, California, United States of America; 2 Departament de Fisica i Enginyeria Nuclear, Universitat Politecnica de Catalunya, Terrassa, Spain; 3 Howard Hughes Medical Institute and Division of Biology, Department of Bioengineering and Applied Physics, California Institute of Technology, Pasadena, California, United States of America; Mount Sinai School of Medicine, United States of America

## Abstract

Under conditions of nutrient limitation, *Bacillus subtilis* cells terminally differentiate into a dormant spore state. Progression to sporulation is controlled by a genetic circuit consisting of a phosphorelay embedded in multiple transcriptional feedback loops, which is used to activate the master regulator Spo0A by phosphorylation. These transcriptional regulatory interactions are “bandpass”-like, in the sense that activation occurs within a limited band of Spo0A∼P concentrations. Additionally, recent results show that the phosphorelay activation occurs in pulses, in a cell-cycle dependent fashion. However, the impact of these pulsed bandpass interactions on the circuit dynamics preceding sporulation remains unclear. In order to address this question, we measured key features of the bandpass interactions at the single-cell level and analyzed them in the context of a simple mathematical model. The model predicted the emergence of a delayed phase shift between the pulsing activity of the different sporulation genes, as well as the existence of a stable state, with elevated Spo0A activity but no sporulation, embedded within the dynamical structure of the system. To test the model, we used time-lapse fluorescence microscopy to measure dynamics of single cells initiating sporulation. We observed the delayed phase shift emerging during the progression to sporulation, while a re-engineering of the sporulation circuit revealed behavior resembling the predicted additional state. These results show that periodically-driven bandpass feedback loops can give rise to complex dynamics in the progression towards sporulation.

## Introduction

Terminal differentiation processes are critical throughout cell biology. Examples in eukaryotes include neuron development [1], maturation of *Xenopus* oocytes [2], cell death by apoptosis [3], meiosis in yeast [4] and flowering in plants [5]. Despite significant effort towards the identification of the molecular circuitry controlling such processes, it often remains unclear how the approach to a terminal state plays out dynamically at the level of individual cells. One of the best-studied terminal differentiation processes is sporulation of the bacterium *Bacillus subtilis*, through which a vegetative cell under nutritional stress transforms into a stable, dormant spore [6]. In some conditions, progression towards the terminal state spans several cell cycles, after which a precise sequence of molecular events remodels the cell into a spore [7]–[9]. Although much of the genetic circuit regulating sporulation initiation is known, it is still unclear how cells control the sequence of events leading to differentiation.

**Figure 1:**
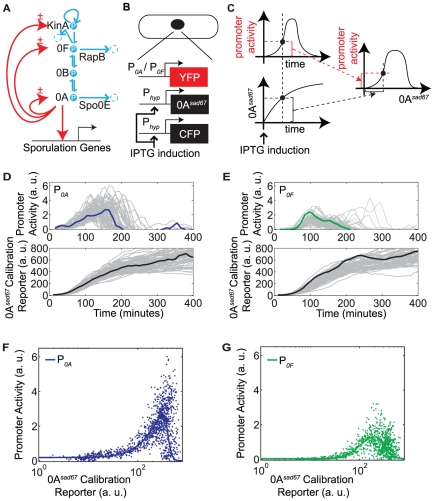
Transcriptional bandpasses in the sporulation initiation circuit. A. Diagram of the sporulation initiation circuit in *B. subtilis*. The main phosphorelay is embedded inside multiple transcriptional feedback loops (red arrows, the ± symbol indicates bandpass regulation). Kinase autophosphorylation, phosphotransfers, and phosphatase activities are shown in blue arrows. RapB and Spo0E are examples of phosphatases that remove phosphate from the indicated proteins. B–C. Schematic of the strains and the method used to measure the bandpass functions of P*_0A_* and P*_0F_*. D–E. Single-cell measurements of promoter activities of P*_0A_* (N = 75) and P*_0F_* (N = 64) (top panels), and corresponding induction profiles of the Spo0A*^sad67^* calibration reporter (bottom panels). Individual traces are shown in gray. A specific trace in each plot is highlighted in colored line. F–G. Single-cell measurements represented as P*_0A_*/P*_0F_* promoter activity versus the Spo0A*^sad67^* calibration reporter (dots). Solid lines show a fit resulting from the following set of parameter values: 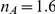, 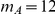, 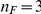, 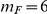, 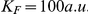, 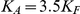, 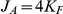, 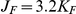, 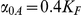, 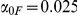, 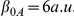, 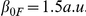.

*Bacillus subtilis* cells control sporulation by modulating the expression and phosphorylation of the master transcription factor Spo0A. The phosphorylation of Spo0A is controlled by a four-component phosphorelay, while its expression is controlled by Spo0A itself, as well as other regulators, some of which are also under the direct or indirect control of Spo0A (Fig. 1A) [10]–[13]. The inputs to this signaling circuit are five sporulation kinases, KinA-KinE (for simplicity, only KinA is shown in the figure), which autophosphorylate in response to nutrient limitation and other stresses, allowing them to transfer phosphates to Spo0F, which are then reversibly relayed via Spo0B to the master regulator Spo0A [10]. Additionally, Spo0F and Spo0A are dephosphorylated by the Rap and Spo0E family of phosphatases, respectively [11]–[13]. The KinA-KinE kinases can also act as phosphatases for Spo0F [14], [15]. The phosphorylated form of Spo0A, denoted Spo0A∼P, controls the expression of *kinA*, *spo0F* and *spo0A* itself (but not *spo0B*) (Fig. 1A), forming several feedback loops, which could be critical for the all-or-none nature of sporulation initiation [16].

The feedback loops in this sporulation circuit have three striking features: First, the activities of the promoters controlling *spo0A*, *spo0F*, and *kinA* (referred to as P*_0A_*, P*_0F_* and P*_kinA_* in what follows) respond in a “bandpass” manner to varying concentrations of Spo0A∼P. That is, they are activated by low levels of Spo0A∼P, and repressed by high levels of Spo0A∼P [17], similar to the type of regulation shown to occur in the P*_RM_* promoter of phage lambda in response to CI [18]. Second, as discussed in the model below, there is an additional post-translational “bandpass” regulatory effect due to the dual role of Spo0F, which is required for Spo0A phosphorylation but can also lead to Spo0A dephosphorylation, due to reverse phosphotransfer and the activity of Spo0F phosphatases. These effects can cause net phosphorylation of Spo0A to first increase, and then decrease, as Spo0F levels rise [19]. Third, gene expression during progress to sporulation occurs in a pulse-like fashion every cell cycle [7], [9]. In particular, Spo0A's target promoters, including P*_0A_* and P*_0F_*, pulse once per cell cycle, implying a periodic modulation of the phosphorelay activity, possibly driven by modulation of kinase activity. As a result of these features, models based on continuous, monotonic interactions between components are inadequate to explain the dynamic behavior of this system.

Here we ask how these bandpass and pulsatile features of the phosphorelay circuit affect the approach to sporulation in individual cells. We address this question through a combination of mathematical modeling and single-cell monitoring via time-lapse fluorescence microscopy. Our data show that the bandpass input functions of P*_0A_* and P*_0F_* are shifted relative to each other. A simple mathematical model incorporating the constraints described above (transcriptional bandpasses plus discrete pulses in kinase activity) predicted the additional post-translational bandpass regulation of Spo0A∼P activity by Spo0F expression level. The model also predicted the appearance of a delayed phase shift between pulses of P*_0A_* and P*_0F_* activity, after being in phase for several cell cycles. Our experiments verified the existence of this delayed phase shift. Further analysis of the model indicated the possibility of an additional state in which no phase shift appeared in these promoter activity pulses. Consistent with this prediction, strains containing extra copies of *spo0F* showed additional steady-state-like behavior with no phase shift between P*_0A_* and P*_0F_* pulses. Together, these results provide insight into the interplay between the periodic input to the sporulation circuit and the dynamics of its components.

## Results

### Transcriptional feedback regulation exhibits a characteristic bandpass dependence on Spo0A∼P

In order to characterize the feedback dynamics of the sporulation circuit, we first investigated the quantitative dependence of the activity of the *spo0A* and *spo0F* promoters, denoted P*_0A_* and P*_0F_*, respectively, on Spo0A∼P levels. To do so, we took advantage of the Spo0A*^sad67^* allele, which is known to transcriptionally regulate target promoters without the need for phosphorylation [20], [21]. We constructed two strains (Fig. 1B) in which Spo0A*^sad67^* controlled expression of the yellow fluorescent protein, YFP, from either the P*_0A_* or the P*_0F_* promoter. In both strains, Spo0A*^sad67^* was expressed from the IPTG-dependent P*_hyperspank_* inducible promoter, denoted P*_hyp_* [20] . These strains also incorporated a second copy of P*_hyp_* driving expression of cyan fluorescent protein, CFP, for calibration.

At the start of the experiment, each of the two strains was induced with the same amount of IPTG, and imaged over time using time-lapse microscopy (Fig. 1C). As Spo0A*^sad67^* expression levels increased towards their steady state value, we quantified the activity of the downstream promoters, determined by the rate of increase in YFP fluorescence (see Materials and Methods), as well as the mean fluorescence of the calibration reporter (Fig. 1D–E) [8], [22]–[25]. Then, we plotted these P*_0A_* and P*_0F_* promoter activities against the level of the calibration reporter (and thus of Spo0A*^sad67^*) from the same cell (Fig. 1F–G). For both promoter reporters, these measurements were performed on cells growing in similar microenvironments and fluorescent illumination conditions.

These data provided a quantitative measurement of the bandpass input functions for P*_0A_* and P*_0F_* (Fig. 1F–G). Despite variability, we found that the two promoter reporters displayed both broadly similar features and systematic differences. Thus, although they exhibited coincident pulses of activity, P*_0A_* exhibited greater basal fluorescence expression at low Spo0A*^sad67^* levels, a higher total fluorescence expression level, and a sharper shutoff at high Spo0A*^sad6^* ^7^ levels, compared to P*_0F_*.

To gain insight into the origins of the observed variability, we compared the difference between a cell and its sister cell with the difference between the same cell and a randomly chosen surrogate sister cell (Fig. S1). Here, the difference metric for two given traces is the cumulative sum in time of the absolute difference between them. The difference between sister cells was significantly smaller than that between surrogate sister cells (Fig. S1), suggesting that variable features can be inherited between cell generations.

In order to incorporate the measured bandpass functions into a model of the phosphorelay circuit, we modeled the experimental data using standard promoter activity rate functions that incorporate both activation and repression (solid lines, Fig. 1F–G):
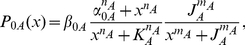
(1)

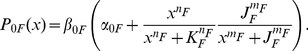
(2)Here, *x* represents [Spo0A∼P], while the *K_i_* and *J_i_* parameters represent, respectively, the activation and repression thresholds of the transcriptional bandpasses. A heuristic fit of this model to the data is shown in Fig. 1F–G.

Together, these results show that P*_0A_* and P*_0F_* encode similarly shaped bandpass functions, but with different quantitative parameters.

### Simple model of phosphorelay predicts post-translational Spo0F bandpass

Next we constructed a simple mathematical model of the phosphorelay circuit based on its phosphorylation, dephosphorylation and phosphotransfer reactions. These reactions are modeled using ordinary differential equations based on standard mass action kinetics. The model consists of four equations representing the phosphorylated forms of the phosphorelay proteins KinA 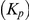, Spo0F 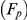, Spo0B 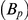, and Spo0A 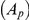,
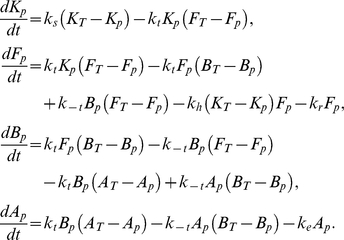
Here, 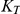, 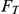, 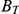, and 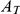 represent total levels of the phosphorelay proteins, 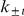 is the rate of forward/backward phosphotransfer rates in the phosphorelay, 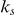 is the rate of autophosphorylation of the kinase, 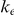 and 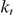 are the dephosphorylation rates of Spo0A and Spo0F by their phosphatases, respectively, and 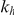 is the rate of dephosphorylation of Spo0F by KinA. The parameter values used for this set of equations are listed in Table 1. The choice of these parameter values was guided by the notion that reactions mediating phosphate flux are typically faster than the cell-cycle timescale 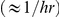. The rate constants for the bimolecular reactions in the model (see Table 1) are similar to effective rate constants for the phosphotransfer reactions estimated from *in vitro* experiments [26].

**Table 1:** Parameters for the phosphorylation, phosphotransfer, and dephosphorylation reactions of the phosphorelay circuit.

Parameter	Value
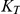	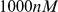
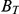	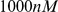
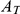	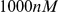
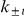	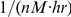
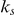	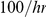
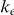	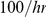
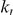	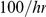
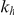	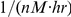

**Figure 2:**
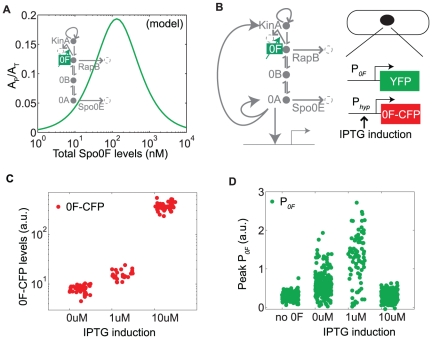
Phosphorelay activity depends on total Spo0F levels in a bandpass manner. A. Fraction of phosphorylated Spo0A computed for different Spo0F levels in the phosphorelay model. B. Diagram of the sporulation circuit and the strain schematic used to test the post-translational bandpass prediction (compare to Fig. 1A). C. As the amount of inducer IPTG is increased (0 uM, 1 uM, 10 uM), there is an increase in Spo0F induction level. D. Peak P*_0F_* pulse amplitude over time for different IPTG induction levels has a bandpass shape. There is some activity even at 0 uM IPTG possibly due to basal Spo0F expression, although it is too low to induce sporulation.

Using this model, we first computed the response of 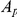 to different Spo0F expression levels and observed a bandpass response (Fig. 2A). Removal of reverse phosphotransfer from the model 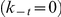 completely abolishes the inhibition of 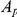 by high Spo0F levels (Fig. S2A, C), showing that reverse phosphotransfer is necessary for this bandpass response. Similarly, when the Spo0F phosphatase activity is set to zero in the model 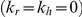, high levels of Spo0F do not inhibit 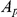 (Fig. S2B, C). This bandpass response is also seen in a more realistic model of the phosphorelay circuit that includes cell-cycle-dependent pulsing and transcriptional feedbacks (Fig. S2D–G), as well as in a more complicated reaction scheme of the core phosphorelay (Text S1, Fig. S2H). Thus, the bandpass response of Spo0A phosphorylation to Spo0F levels in this model is a direct result of reversibility of the phosphotransfer, which allows phosphates to flow backwards from Spo0A to Spo0F, where they can be hydrolyzed by Spo0F phosphatases and lost from the system.

### Experiments confirm the post-translational bandpass of phosphorelay activity

The prediction that Spo0F levels have a bandpass effect on Spo0A activity is in qualitative agreement with previous experimental results [19]. To measure the post-translational bandpass at the level of single cells, we induced Spo0F to different levels using a Spo0F-CFP protein under the IPTG-controlled promoter P*_hyp_* in a strain where the endogenous copy of *spo0F* was deleted (Fig. 2B–C). We quantified the resulting activity of Spo0A∼P by measuring the peak amplitude of the P*_0F_*-YFP pulses. We observed a striking bandpass response similar to that predicted by the model (Fig. 2D). The physiological response of sporulation was inhibited at the highest IPTG induction (results not shown), indicating that the bandpass response is due to low Spo0A∼P and not an artifact of the transcriptional bandpass in the P*_0F_* reporter.

### Dynamical model driven by KinA pulses and incorporating transcriptional bandpasses predicts emergence of a delayed phase shift between sporulation components

**Figure 3:**
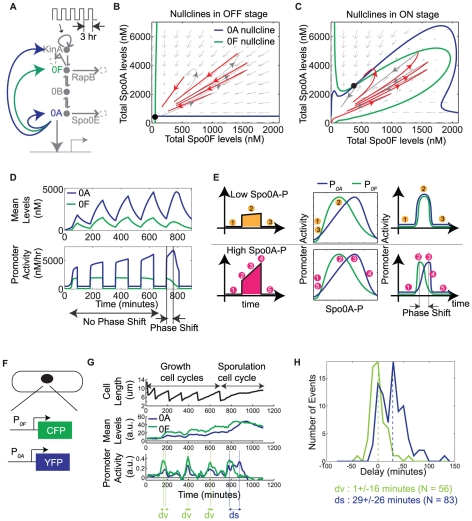
Delayed phase shift emerges in the periodic activity of phosphorelay genes *spo0A* and *spo0F*. A. Diagram of the phosphorelay circuit driven by square wave pulses in kinase autophosphorylation. B–C. Phase portraits computed from the reduced model for “ON” and “OFF” parts of the square wave: Solid lines represent nullclines of Spo0A (blue) and Spo0F (green), arrows depict the slope field, and black circles denote stable steady states. A red-gray color-code is used to plot the trajectory on each phase plane, with red marking the portion of the trajectory that evolves on the active phase plane, and gray marking the portion on the other phase plane. D. Mean levels of Spo0A and Spo0F (top panel) computed from the complete model, and their corresponding promoter activities (bottom panel). E. Illustration of the delayed phase shift as a mapping from a Spo0A∼P pulse to the transcriptional bandpasses, and from these bandpasses to the promoter activity pulses. Orange circles (top row, 1-2-3) label time-points before, during, and after a low amplitude Spo0A∼P pulse. Pink circles (bottom row, 1-2-3-4-5) label time-points before, during, and after a high amplitude Spo0A∼P pulse. Colored circles are placed at corresponding points on the transcriptional bandpasses and promoter activity pulses. F. Schematic of the two-color strain used to experimentally measure the single-cell dynamics of P*_0A_* and P*_0F_*, which are fused to YFP and CFP fluorescent reporters, respectively. G. Single-cell measurements of P*_0A_* and P*_0F_* promoter activities (bottom panel), mean levels of YFP and CFP (middle panel), and the cell length (top panel). “dv” and “ds” denote the time difference between the P*_0A_* and P*_0F_* peak pulse amplitudes in the pre-sporulation vegetative growth cycles and in the sporulation cycle, respectively. H. Experimentally measured distribution of the P*_0A_*-P*_0F_* time differences in pre-sporulation vegetative growth cycles and in sporulation cycles.

Next, we incorporated the cell-cycle-dependent pulsatile regulation of phosphorelay activity into the model. For simplicity, we assumed that the kinase autophosphorylation rate is a square wave 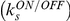, with a period fixed at 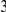 hours, similar to the cell-cycle times observed experimentally (Fig. 3A). We further assumed, arbitrarily, that the duration of the “ON” phase spanned 50% of that period. We also included in the model the feedbacks on the phosphorelay proteins based on the transcriptional bandpass measurements. In this more complete model, the total level of each phosphorelay protein can increase due to transcription and decrease by degradation. While both phosphoforms can be degraded, we assume that transcription creates only unphosphorylated proteins. These reactions are also modeled using ordinary differential equations. Because there are no known active degradation processes for these proteins, we assume that the only source of degradation is dilution by cell growth, which is modeled as a first-order decay process with rate constant 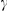. With these assumptions, we obtain the following equations for the total concentrations of the phosphorelay proteins KinA, Spo0F, Spo0B, and Spo0A, denoted 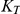, 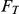, 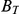, and 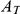, respectively:
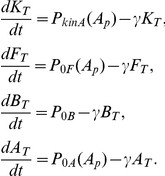
Here we assume that protein degradation is negligible compared to protein dilution. Hence, 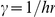, similar to the rates of dilution by cell growth observed experimentally. This degradation term is also added to the equations of the phosphorylated proteins. The mean rates of expression from the P*_0F_* and P*_0A_* promoters, as functions of Spo0A∼P 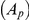, are denoted 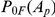 and 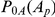, respectively, and are based on the empirically determined bandpass functions, given by Eqs. (1)–(2). The bandpass parameters are constrained by the experimental measurements above, with the free parameter 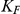 chosen to be 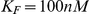, similar to values of Spo0A∼P's DNA binding affinity estimated from previous experiments [27]. Additionally, because previous results suggest that the promoter of KinA is also a bandpass like Spo0A [17], we assume that, 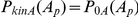 (the importance of this assumption is checked in the dynamical analysis described below). There is no known transcriptional regulation of Spo0B by Spo0A∼P. Moreover, experimental measurements show that expression level of a fluorescent reporter fused to P*_0B_* is already turned on prior to the beginning of progression to sporulation, and that this reporter changes less than twofold during sporulation initiation (Fig. S3). Consequently, we assume 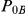 to be constant, 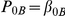. The maximal expression strengths of these promoters are free parameters and are set to 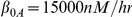, 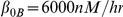, 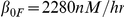. The parameters used in this set of equations are summarized in Table 2.

**Table 2:** Parameters for the periodic input and the production-degradation reactions of the phosphorelay circuit.

Parameter	Value
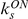	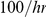
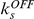	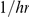
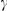	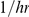
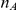	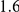
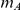	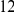
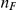	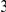
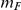	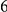
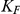	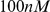
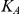	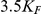
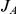	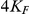
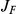	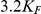
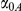	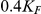
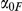	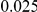
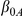	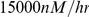
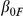	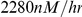
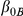	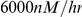

In order to understand the implications of the bandpass regulation on the dynamics of the phosphorelay in cells, we first set out to simplify the model by reducing its dimensionality. The model has eight dimensions: the expression levels of the four proteins and their phosphorylation states. However, because the timescale of phosphorylation is much faster than that of protein production and degradation, the four degrees of freedom corresponding to phosphorylation can be adiabatically eliminated. In addition, since 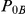 is constant, 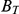 can be set to a fixed value. Third, having assumed 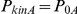, KinA and Spo0A protein levels are represented by the same degree of freedom. These considerations allow us to reduce the model to two effective dimensions, one for total Spo0A and another one for Spo0F:
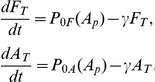
In this reduced model, the promoter activities P*_0A_* and P*_0F_* are functions of 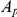, which itself is a function of the values of the total levels of the phosphorelay proteins and the input square wave. This dependence is computed from the equations of the phosphorylated proteins (see Materials and Methods).

To analyze the dynamic behavior of this two-dimensional model, we computed the nullclines 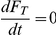 and 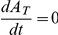, on the Spo0A-Spo0F phase plane. The trajectories in the two-dimensional model switch between two phase planes, corresponding to the “ON” and “OFF” parts of the square wave (Fig. 3B–C). For the parameters chosen, the nullclines cross at a single point in both phase planes, which is a stable steady state. Consider the trajectory starting near the origin, where levels of phosphorelay proteins are low (gray and red lines in Fig. 3B–C). Such a trajectory spends the first few periods traversing up and down a single line passing through the origin, for which Spo0A and Spo0F are proportional to (i.e. in phase with) each other, and then curves away from this line (see Fig. 3B–C). At that point the proportionality between Spo0A and Spo0F breaks down, leading to the appearance of a phase shift between the activities of the P*_0A_* and P*_0F_* promoters (Fig. 3D). The assumption that 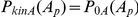
does not qualitatively change the dynamical picture described here (Fig. S4).

The emergence of this delayed phase shift from the model can be understood in terms of the effect on the transcriptional bandpass functions of P*_0A_* and P*_0F_* (Fig. 3E). In the initial periods of the square wave, the peak amplitude of Spo0A∼P pulse is low and it accesses only the activating parts of the promoter input functions. Consequently, the pulses in P*_0A_* and P*_0F_* are proportional to each other. In the last period, the peak amplitude of Spo0A∼P is higher and it sweeps across the bandpass region. When this happens, there is a time interval in which P*_0F_* is repressed while P*_0A_* is activated, generating a phase shift between the P*_0A_* and P*_0F_* pulses. The duration of this phase shift is determined by the rate of increase in Spo0A∼P level, with a faster rate of increase resulting in a smaller duration. In the limit that Spo0A∼P levels reach their final value instantaneously, there is no phase shift.

### Delayed phase shift between P*_0A_* and P*_0F_* can be observed experimentally

To verify experimentally the existence of this delayed phase shift between the activities of P*_0A_* and P*_0F_*, we constructed a two-color strain by which the two promoter activities could be simultaneously measured in the same cell (Fig. 3F). Both P*_0A_* and P*_0F_* pulsed in the sporulation cycles as well as in the pre-sporulation growth cycles (Fig. 3G). We found that the distribution of delays between P*_0A_* and P*_0F_* pulse peaks in the pre-sporulation growth cycles (Fig. 3H) is close to zero (1±16 min, N = 56), i.e. the promoters pulse in phase, while those in the sporulation cycle are significantly larger than 0 (29±26 min, N = 83; KS test, p<10^−7^).

### Bandpass dynamics can stabilize an alternate cellular state

**Figure 4:**
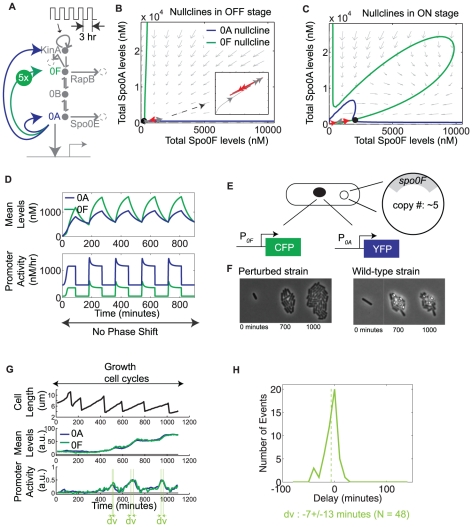
Increase in *spo0F* copy number leads to a non-sporulating alternate state with no phase shift. A. Diagram of the phosphorelay circuit with increased *spo0F* copy number. B–C. Phase portraits computed from the reduced model with increased *spo0F* copy number for the “ON” and “OFF” parts of the square wave. D. Mean levels of Spo0A and Spo0F (top panel) computed from the complete model, and their corresponding promoter activities P*_0A_* and P*_0F_* (bottom panel). E. Schematic of the strain used to experimentally test the alternate state prediction. F. Frames from time-lapse movies showing that sporulation in the perturbed strain is inhibited in comparison to the wild-type strain. G. Single-cell measurements of *P_0A_* and *P_0F_* promoter activities (bottom panel), mean levels of YFP and CFP (middle panel), and the cell length (top panel). “dv” denotes the time difference between the P*_0A_* and P*_0F_* peak pulse amplitudes in the pre-sporulation vegetative growth cycles. H. Experimentally measured distribution of the P*_0A_*-P*_0F_* time-differences in pre-sporulation vegetative growth cycles.

Nullcline analysis of the simplified two-dimensional model is also useful in connecting the delayed phase shift in the phosphorelay dynamics with the post-translational bandpass (Fig. 2A). In the “ON” phase plane, an increase in *spo0F* copy number (Fig. 4A) shifts the Spo0F nullcline to the right, which leads to a low 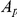 fixed point (Fig. 4B–C). In this situation, pulses of KinA activity lead to trajectories in phase space for which Spo0A and Spo0F are proportional for the duration of the square wave input. The promoter activities P*_0A_* and P*_0F_* also maintain their proportionality, and so exhibit no phase shift (Fig. 4D). Thus, the model predicts that an increase in *spo0F* copy number should lead to an alternate behavior with low levels of Spo0A∼P, where P*_0A_* and P*_0F_* pulse permanently in phase with each other.

In terms of the representation in Fig. 3E, this perturbation in *spo0F* copy number restricts Spo0A∼P activity to low amplitude pulses. Since low amplitude Spo0A∼P pulses can give rise to pulses in P*_0A_* and P*_0F_*, this perturbed trajectory is similar to the initial periods of the square wave where P*_0A_* and P*_0F_* pulses are in phase (Fig. 3D). However, these initial periods are limited in number, whereas in the perturbed system, the phase shift never appears (Fig. 4D).

To investigate the dependence of the alternate state on the two types of bandpasses in the model, we examined the changes to the nullclines in response to individual bandpass perturbations. In the absence of reverse phosphotransfer, a feature needed for the post-translational bandpass, there is no alternate state, but the nullclines are strongly perturbed and change their orientation (Fig. S5A, B). When the activation and repression parts of the P*_0F_* transcriptional bandpass are removed individually, the nullclines are perturbed so that the alternate state exists (Fig. S5A, C–D). In comparison to this, when the activation and repression parts of the P*_0A_* transcriptional bandpass are removed individually, the nullclines are perturbed but without the appearance of the alternate state (Fig. S5A, E–F). So, to a first approximation, it is the coupling of the post-translational bandpass and the P*_0F_* transcriptional bandpass that appears to enable the alternate state. Further analysis will be required to map regions in parameter space where the alternate state behavior exists.

To experimentally test the predicted effects of increased *spo0F* copy number, we first cloned the entire *spo0F* gene into pHP13, a plasmid with copy number of ∼5 [24]. We transformed this plasmid into the two-color P*_0A_*-P*_0F_* reporter strain (Fig. 4E), and used it to measure the phase shift between P*_0A_* and P*_0F_*. The distribution of delays between P*_0A_* and P*_0F_* pulse peaks (−7±13 minutes, N = 48) indicates that their activities are in phase, consistent with the model (Fig. 4G–H). Moreover, an additional, indirect line of evidence suggests that cells are in a state resembling the low Spo0A∼P alternate state: The level of Spo0A∼P in these cells is not high enough to initiate sporulation (Fig. 4F), but is high enough to elicit pulses in P*_0A_* and P*_0F_* promoter activities (Fig. 4G). Thus Spo0A∼P levels are low, but non-zero. Together, these experimental data qualitatively support the model prediction of an alternate state with higher Spo0F copy number.

## Discussion

The central element of the sporulation circuit in *B. subtilis* is a phosphorelay embedded in bandpass transcriptional feedback loops, which are activated in a pulse-like manner, once per cell cycle. We have investigated the dynamical consequences of this particular architecture through a combination of single-cell monitoring and mathematical modeling. Our mathematical model reveals several striking features of this system, all of which are confirmed experimentally at the single-cell level: First, the response of Spo0A∼P activity has a bandpass dependence on Spo0F protein concentration. Second, pulses in the Spo0A and Spo0F promoter activities are in phase within the initial cell cycles subsequent to stress, but eventually develop a phase shift. Third, an alternate cellular state can be accessed, in which P*_0A_* and P*_0F_* pulse and remain in phase indefinitely, signifying the presence of low amplitude Spo0A∼P pulses. Together, these results show how pulsing together with bandpass-like features in a feedback circuit gives rise to surprisingly complex dynamics in the lead-up to sporulation.

According to our model, the emergence of the delayed phase shift depends on the inter-relationship between the periodic input to the sporulation circuit and the dynamics of the circuit components. In particular, the phase shift appears when the Spo0A∼P pulse amplitude is high enough to access the repressing part of the transcriptional bandpasses. Thus, the rate of increase of Spo0A∼P determines the number of periods needed for the phase shift to appear. Additionally, in a given period, the magnitude of this rate in comparison to the relative duration of the “ON” phase plays a role in determining the maximal value of Spo0A∼P pulse. If the rate is too slow, then Spo0A∼P pulse is shut off before it reaches a value large enough to generate a phase shift, as seen in the earlier periods. Only if the rate is high enough, does the Spo0A∼P pulse rise to a value large enough to generate a phase shift before it is turned off. These are important considerations as the rate of increase of Spo0A∼P determines the duration of the phase shift, and consequently the timing of the sequence of events leading to the transition to sporulation.

An inherent challenge in the model is to map the relationship between perturbations in the transcriptional bandpasses and the sporulation dynamics. Currently, this is hindered by a limited understanding of how Spo0A∼P interacts with the promoter regions of P*_0A_* and P*_0F_* to generate the bandpass mode of regulation. An approach that determines how bandpass regulation is encoded in the promoter architectures will be required to overcome this. This will also be useful in corroborating the parameter estimates reported here or in obtaining more exact ones. In particular, while this study shows that the P*_0A_* and P*_0F_* transcriptional bandpasses are quantitatively different, further studies may be required to determine the exact ratios of the bandpass thresholds and their slopes. More generally, while the parameters for the production-degradation and phosphorylation-dephosphorylation-phosphotransfer used here are reasonable, determining their exact values requires further investigation. Following this, the model can be combined with systematic perturbations to the promoters to develop further insight into the dynamics leading to sporulation initiation.

Studies of terminal differentiation dynamics in individual cells can reveal the fine structure behavior of underlying regulatory circuits. Here, this behavior takes the form of the emergence of a delayed phase shift and the capability, in an alternate state, to suspend the appearance of the phase shift. Fine-structure studies of other circuits regulating terminal differentiation may reveal further instances of temporal order in gene expression. Dynamic single-cell studies of other processes should reveal if constituent genes are sequentially expressed, and if this temporal order is operationally critical or is just a by-product of resource optimization by cells preparing for a terminal state [28].

## Materials and Methods

### Strain Construction

All strains were constructed using standard *B. subtilis* protocols and molecular biology methods (Table 3). The background of all strains used was *B. subtilis* PY79. For image segmentation, a constitutive promoter expressing fluorescent protein reporter RFP was chromosomally integrated into PY79 (LS1). Promoter fusions to fluorescent proteins were chromosomally integrated using *Bacillus* integration vectors pDL30, ECE174 (both from lab stocks), and pER82 (kindly provided by Jonathan Dworkin). We also used the antibiotic-switching plasmid ECE73 (*cmR→neoR*). The integration vector 174 hs (from lab stock) containing the IPTG-inducible LacI system added to the ECE174 backbone was used to induce Spo0F to different levels. For copy number perturbation, the plasmid pHP13 (from lab stock) was used. Based on these, other plasmids for promoter fusions and for circuit perturbations were constructed with *Escherichia coli* DH5α or DH5αZ1 by using standard methods of PCR, restriction enzyme digests, and ligations (Table 4, 5):

- The plasmid pSS938 was constructed by ligating the EcoRI-BamHI fusion PCR fragment P*_0A_*-YFP and pDL30 cut with EcoRI-BamHI. The fusion PCR fragment P*_0A_*-YFP was made by fusing P*_0A_* (primers oss80967, oss80949 and template PY79) and YFP (primers oss80944, oss80147 from a template plasmid containing YFP from lab stock) using primers oss80967, oss80147.
- The plasmid pSS925 was constructed by ligating the BamHI-EcoRI fusion PCR fragment P*_0F_*-CFP and ECE174 cut with EcoRI-BamHI. The fusion PCR fragment P*_0F_*-CFP was made by fusing P*_0F_* (primers oss80973, oss80972 and template PY79) and CFP (primers oss80975, oss80146 from a template plasmid containing CFP from lab stock) using primers oss100436, oss80146.
- The plasmid pSS711 was constructed by ligating the EcoRI-BamHI PCR fragment *spo0F* (primers oss80907, oss80181 and template PY79) and pHP13 cut with EcoRI-BamHI.
- The plasmid pSS1027 was constructed by ligating the EcoRI-BamHI fusion PCR fragment P*_hyp_*-CFP and ECE174 cut with EcoRI-BamHI. The fusion PCR fragment P*_hyp_*-CFP was made by fusing P*_hyp_* (primers oss100801, oss100805 and template MF2158) and CFP (primers oss100804, oss100802 from a template plasmid containing CFP from lab stock) using primers oss100801, oss100802.
- The plasmid pSS916 was constructed by ligating the EcoRI-BamHI fusion PCR fragment P*_0F_*-YFP and pDL30 cut with EcoRI-BamHI. The fusion PCR fragment P*_0F_*-YFP was made by fusing P*_0F_* (primers oss80973, oss100437 and template PY79) and YFP (primers oss100438, oss80147 from a template plasmid containing YFP from lab stock) using primers oss100436, oss80147.
- The plasmid pSS1025 was constructed by ligating the EcoRI-BamHI fusion PCR fragment P*_0F_*-YFP and pER82 cut with EcoRI-BamHI. The fusion PCR fragment P*_0F_*-YFP was made as above.
- The plasmid pSS1031 was constructed by ligating the HindIII-NheI fusion PCR fragment Spo0F-CFP and 174 hs cut with HindIII-NheI. The fusion PCR fragment Spo0F-CFP was amplified (primers oss100908, oss100905) from a vector already containing a Spo0F-CFP fragment, which was made by fusing the Spo0F gene (primers oss80900, oss80913 and template PY79) and CFP (primers oss80912, oss100802 from a template plasmid containing CFP from lab stock) using primers oss80900, oss100802.
- The *spo0F* deletion PCR was made by fusing the gene conferring *specR* with flanking regions bearing homology upstream and downstream of the Spo0F coding region. The primer pairs (oss80928, oss80924), (oss80922, oss80923) and (oss80925, oss80927) were used to PCR *specR* (pDL30 template), upstream homology region (PY79 template) and downstream homology region (PY79 template), respectively. These pieces were then fused using primers oss80922 and oss80927.

**Table 3:** List of Strains.

Number	Strain	Reference/Source/Construction
MF2158	PY79 *spo0A*::*specR*::*cmR* P*_lacI_*-LacI P*_hyp_*-*0A^sad67^*	Lab stock
LS1	PY79 *ppsB*::*ermR* P*_trpE_*-RFP	Lab stock
LS4	PY79	Lab Stock
SS813	PY79 *ppsB*::*ermR* P*_trpE_*-RFP *spo0A*::*specR*::*cmR* P*_lacI_*-LacI P*_hyp_*-*0A^sad67^*	MF2158→LS1
SS1039	PY79 *sacA*:: *cmR* P*_hyp_*-CFP	pSS1027→LS4
SS1060	PY79 *sacA*:: *cmR*::*neoR* P*_hyp_*-CFP	ECE73→SS1039
SS1075	PY79 *ppsB*::*ermR* P*_trpE_*-RFP *spo0A*::*specR*::*cmR* P*_lacI_*-LacI P*_hyp_*-*0A^sad67^ sacA*:: *cmR*::*neoR* P*_hyp_*-CFP	SS1060→SS813
SS1125	PY79 *ppsB*::*ermR P_trpE_*-RFP *spo0A*::*specR*::*cmR* P*_lacI_*-LacI P*_hyp_*-*0A^sad67^ sacA*:: *cmR*::*neoR* P*_hyp_*-CFP *amyE*::*specR* P*_0A_*-YFP	pSS916→SS1075
SS1017	PY79 *ppsB*::*ermR* P*_trpE_*-RFP *amyE*::*specR* P*_0F_*-YFP	pSS938→LS1
SS1019	PY79 *ppsB*::*ermR* P*_trpE_*-RFP *amyE*::*specR* P*_0F_*-YFP *spo0A*::*specR*::*cmR* P*_lacI_*-LacI P*_hyp_*-*0A^sad67^*	MF2158→SS1017
SS1106	PY79 *ppsB*::*ermR* P*_trpE_*-RFP *amyE*::*specR* P*_0F_*-YFP *spo0A*::*specR*::*cmR* P*_lacI_*-LacI P*_hyp_*-*0A^sad67^ sacA*:: *cmR*::*neoR* P*_hyp_*-CFP	SS1060→SS1019
SS946	PY79 *ppsB*::*ermR* P*_trpE_*-RFP *sacA*::*cmR* P*_0F_*-CFP	pSS925→LS1
SS1007	PY79 *ppsB*::*ermR* P*_trpE_*-RFP *sacA*::*cmR* P*_0F_*-CFP *amyE*::*specR P_0A_*-YFP	pSS916→SS946
SS1021	PY79 *ppsB*::*ermR* P*_trpE_*-RFP *sacA*::*cmR::neoR* P*_0F_*-CFP *amyE*::*specR* P*_0A_*-YFP	ECE73→SS1007
SS1023	PY79 *ppsB*::*ermR* P*_trpE_*-RFP *sacA*::*cmR::neoR* P*_0F_*-CFP *amyE*::*specR* P*_0A_*-YFP pHP13-*spo0F*	pSS711→SS1021
SS745	PY79 *spo0F*::*specR*	*spo0F* deletion PCR→LS4
SS843	PY79 *ppsB::ermR* P*_trpE_*-RFP *spo0F*::*specR*	SS745→LS1
SS1037	PY79 *ppsB::ermR* P*_trpE_*-RFP *spo0F*::*specR amyE*::*neoR* P*_0F_*-YFP	pSS1025→SS843
SS1064	PY79 *ppsB::ermR* P*_trpE_*-RFP *spo0F*::*specR amyE*::*neoR* P*_0F_*-YFP *sacA::cmR* P*_lacI_*-LacI P*_hyp_*-Spo0F-CFP	pSS1031→SS1037

**Table 4:** List of Plasmids.

Number	Plasmid
pSS916	pDL30 P*_0A_*-YFP
pSS925	ECE174 P*_0F_*-CFP
pSS711	pHP13-*spo0F*
pSS1027	ECE174 P*_hyp_*-CFP
pSS938	pDL30 P*_0F_*-YFP
pSS1031	174 hs Spo0F-CFP
pSS1025	pER82 P*_0F_*-YFP

**Table 5:** List of Primers.

Number	Primer Sequence (5′-3′)
oss80146	ATA GAATTC AAAAGGCTGAACCCTAAGGT
oss80147	GAT GGATCC GCAATGATGAACCAGTAAGAGTAGC
oss80967	ACT GAATTC CAGAAGCAGGAATCGATATTTATGG
oss80949	CAACGCCGGTGAACAGTTCTTCACCTTTGCTCAT GTTTCTTCCTCCCCAAATGTAGTTAA
oss80944	GTGAATCCTGTTAACTACATTTGGGGAGGAAGAAAC ATGAGCAAAGGTGAAGAACTGTTC
oss80973	GGCCTGCTGGTAATCGCAGGCCTTTTTATT AATCCTCCTTTATAACGTACAATATCAGTA
oss100436	CAC GGATCC GGCCTGCTGGTAATCGCAGGCCTTTTTATTAATCCTCCTTTATAACGTACA
oss80972	AACTCCAGTGAAAAGTTCTTCTCCTTTACGCAT ATTCATCATTTTACACCCCAATATTAT
oss80975	CGAAAATCATAATATTGGGGTGTAAAATGATGAAT ATGCGTAAAGGAGAAGAACTTTTCA
oss80907	AGC GGATCC AAGTGAATCCTCCTTTATAACGTACAATA
oss80181	ATA GAATTC GTCAGTTAGACTTCAGGGGCAGAT
oss100801	TAT GAATTC GACTCTCTAGCTTGAGGCATCAAATAA
oss100805	AACTCCAGTGAAAAGTTCTTCTCCTTTACGCAT AGTAGTTCCTCCTTATGTGTCGACTAA
oss100804	AATTAAGCTTAGTCGACACATAAGGAGGAACTACT ATGCGTAAAGGAGAAGAACTTTTCA
oss100802	ATA GGATCC AAAAGGCTGAACCCTAAGGT
oss100436	CAC GAATTC GGCCTGCTGGTAATCGCAGGCCTTTTTATT AATCCTCCTTTATAACGTACA
oss100437	AACGCCGGTGAACAGTTCTTCACCTTTGCTCAT ATTCATCATTTTACACCCCAATATTAT
oss100438	ACGAAAATCATAATATTGGGGTGTAAAATGATGAAT ATGAGCAAAGGTGAAGAACTGTTC
oss100908	ATA AAGCTT ACATAAGGAGGAACTACT ATGATGAATGAAAAAATTTTAATCGTTG
oss100905	ATA GCTAGC AAAAGGCTGAACCCTAAGGT
oss80900	ATA GAATTC AGTGAATCCTCCTTTATAACGTACAATAT
oss80913	TGAAAAGTTCTTCTCCTTTACGCATGTTAGACTTCAGGGGCAGATATTTT
oss80912	AAAATATCTGCCCCTGAAGTCTAAC ATGCGTAAAGGAGAAGAACTTTTCA
oss80922	TATCAGGATGAAGTGGTGTACGAGC
oss80927	TAACTGTTTCTTTGATCGCTTCACG
oss80924	CGAAAATCATAATATTGGGGTGTAAACATATGCAAGGGTTTATTGTTTTCTAA
oss80923	TTAGAAAACAATAAACCCTTGCATATGTTTACACCCCAATATTATGATTTTCG
oss80925	ATGTATTCACGAACGAAAATCGATCAAAAAGAAGAAACAAATGAATCATG
oss80928	CATGATTCATTTGTTTCTTCTTTTTGATCGATTTTCGTTCGTGAATACAT

### Time-lapse Microcopy

Cells to be imaged were inoculated in CH media and grown overnight a 30°C shaking water incubator. They were then diluted 1∶40 into fresh CH media and grown for 2–3 hours so that the OD600 was in the range 0.5–0.7. Following this, they were resuspended 1∶1 in Sterlini-Mandelstam (SM) media after two washes in SM. 0.5 ul of this was spotted on an appropriate pad (0.5 mm×0.5 mm), allowed to dry, and flipped onto a glass-bottom dish (Wilco). The dish was sealed with parafilm to reduce pad evaporation during imaging. The pads were made from SM media mixed with 1.5% Low Melting Point Agarose (Omni). Required amount of inducer IPTG was added to the pads.

Progression to sporulation was imaged at 37°C using a microscope automated for time-lapse fluorescence data acquisition. Images were acquired every 10 minutes on a Nikon Eclipse-Ti inverted microscope fitted with a perfect focus system, ASI motorized stage, Photometrix Coolsnap HQ2 camera, Sutter Lambda LS Xenon Arc lamp, and controlled from a computer using MetaMorph.

### Data Analysis

The fluorescence values and lengths in single cells were extracted with customized segmentation and tracking algorithms coded in MATLAB and C. Promoter activity per unit length 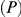 was calculated from the measured mean fluorescence value 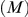 and cell lengths 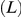 using the formula:
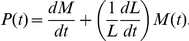



The derivative was computed from a smoothened version of the mean fluorescence and cell length. For this, mean fluorescence was smoothened with a moving average filter and length was fitted to a second order polynomial. To plot the transcriptional bandpass characteristics, time traces up to the initiation of sporulation, as seen from the appearance of a fluorescent dark spot at the tip of the cell, were used. For the post-translational bandpass, the peak P*_0F_* pulse amplitude was measured from T = 0 minutes to T = 600 minutes, and the steady state Spo0F-CFP level was measured at T = 500 minutes. For measurements of phase shift between P*_0A_* and P*_0F_*, promoter activity pulses with peak amplitude greater than a threshold (0.1a.u.) were used.

### Model Analysis

Mathematical modeling was done in MATLAB. Nullclines in the two-dimensional model were computed from the zero contour of the surface functions 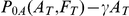 and 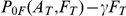. Ordinary differential equations were solved in MATLAB using integrator ode23s. The dependence of Spo0A∼P on Spo0F levels in the model without feedback and pulsing was computed by simulating the equations for a time longer than the timescale of the system. In the simulation of the two-dimensional model, 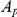 was computed at each timestep by simulating the equation of the phosphorylated proteins using the current values of 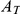, 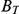, 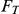, 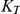, and 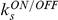.

## Supporting Information

Text S1 Supplementary information describes a model of core phosphorelay with a more complicated reaction scheme that that described in the main text.
(PDF)

Figure S1
**Variability between sister cells is typically smaller than between randomly chosen sister cell pairs.** Difference between a cell and its sister and between the same cell and a randomly chosen surrogate sister cell is calculated for traces of P*_0A_* (A), Spo0A*^sad67^* calibration reporter corresponding to the P*_0A_* bandpass measurement (B), P*_0F_* (C), and Spo0A*^sad67^* calibration reporter corresponding to the P*_0F_* bandpass measurement (D). Each dot represents one cell, and has co-ordinates 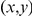, where 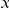 is the difference between sister cells and 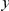 is the difference between surrogate cells. Difference metric for two given traces 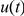 and 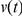 is, 
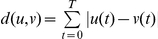
, where 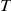 is the minimum duration between the two traces 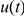 and 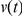. In each case, most points lie above the straight line 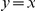, indicating that the difference between sister cells is smaller than between surrogate sister cells.(PDF)

Figure S2 **Post-translational bandpass in the model is due to drain of Spo0A phosphates via reverse phosphotransfer and Spo0F phosphatases.** A–C. Diagrams of the core phosphorelay with no reverse phosphotransfer (A, red) and no Spo0F phosphatase (B, blue), for which the post-translational Spo0F bandpass computation is performed from the simple phosphorelay model (no pulsing or transcriptional feedbacks). Both curves are plotted in C, along with the curve from Fig. 2A (green). D–G. Diagrams of the phosphorelay circuit (D) and versions with no reverse phosphotransfer (E, red) and no Spo0F phosphatase (F, blue), for which the post-translational Spo0F bandpass computation is performed from the full model described in the main text (including pulsing and transcriptional feedbacks, Fig. 3). Peak P*_0F_* pulse amplitudes are plotted for different Spo0F induction levels (at steady state) for three cases: base parameters (G, green), no reverse phosphotransfer (G, red), and no Spo0F phosphatase (G, blue). The absence of Spo0F phosphatases shifts the repression threshold to significantly higher Spo0F values, representing drain of Spo0F phosphates by dilution due to cell growth. H. Green curve is the post-translational Spo0F bandpass computed from a more complicated version of the core phosphorelay (Text S1). The computation is repeated in the absence of reverse phosphotransfer (red) and Spo0F phosphatases (blue).
(PDF)

Figure S3 **Mean fluorescence of a P** ***_0B_*** **reporter changes less than twofold during sporulation initiation.** A. Schematic of the strain with a YFP fluorescent reporter fused to P*_0B_*. The strain uses the plasmid ECE174 P*_0B_*-YFP (from lab stocks) integrated into an LS1 background. Imaging was done as described in the methods section, but on an Olympus IX-81 inverted microscope fitted with an ASI motorized stage, Hamamatsu Orca-ER camera, Sutter Lambda LS Xenon Arc lamp, and controlled using a combined Visual Basic-ImagePro software. B. Mean fluorescence levels are non-zero at the start and change less than twofold during sporulation initiation.
(PDF)

Figure S4
**Qualitative dynamical picture under the P** ***_kinA_*** **= P** ***_0A_*** **assumption is similar to the alternate P** ***_kinA_*** **= P** ***_0F_*** **assumption.** Nullclines in the “ON” stage in the case when 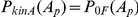, are similar to the case discussed in the main text with the assumption that 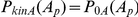, suggesting that this assumption is justified. These nullclines are plotted for same parameters as used in the main text with 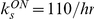.(PDF)

Figure S5 **Effect of bandpass characteristics on the alternate state.** The effect of bandpass characteristics on the alternate state are investigated by comparing “ON” phase planes in the following cases, A. Both P*_0A_* and P*_0F_* bandpass, and reverse phosphotransfer (wild-type case from Fig. 3C, considered in the main text) B. Both P*_0A_* and P*_0F_* bandpass, and NO reverse phosphotransfer: Transcriptional bandpasses remain the same as case A, but there is no post-translational bandpass. Nullclines orient differently from case A, so that the alternate state doesn't exist and its effect on dynamics is minimal. C. P*_0A_* bandpass, P*_0F_* lowpass (only repression), and reverse phosphotransfer: Nullclines are slightly perturbed from case A, with the appearance of a stable steady state, an unstable steady state (white circle), and the associated separatrix. The separatrix is computed by integrating equations backward in time starting from an initial condition near the unstable steady state. The additional stable steady state is similar in location to the alternate state. D. P*_0A_* bandpass, P*_0F_* highpass (only activation), and reverse phosphotransfer: Nullclines are slightly perturbed from case A, with the stable steady state situated like the alternate state. E. P*_0F_* bandpass, P*_0A_* lowpass (only repression), and reverse phosphotransfer: Nullclines are slightly perturbed from case A, but without significant change in the steady state location. F. P*_0F_* bandpass, P*_0A_* highpass (only activation), and reverse phosphotransfer: Nullclines are slightly perturbed from case A, but without significant change in the steady state location. In this case, the stable steady state is situated above the upper Y-axes limit.
(PDF)
