# Supplementary material for: Dynamical Consequences of Bandpass Feedback Loops in a Bacterial Phosphorelay
Source: PLoS One. 2011 Sep 29;6(9):e25102. doi: 10.1371/journal.pone.0025102 (PMC3182994; doi:10.1371/journal.pone.0025102)
Supplement: Text S1 — Supplementary information describes a model of core phosphorelay with a more complicated reaction scheme that that described in the main text. (PDF) [file pone.0025102.s001.pdf]

Post-translational Spo0F bandpass in a more complex model of the core phosphorelay. The post-translational Spo0F bandpass discussed in the main text is based on a simple model of the core phosphorelay. In principle, a more complicated reaction scheme involving reaction intermediates is also possible. Here, we verify the post-translational Spo0F bandpass results in a model of the core phosphorelay that contains these reaction intermediates. This reaction schemes has been previously used for phosphorelay-like signaling systems [1].

This model consists of 17 variables, of which eight correspond to the phosphoforms of the four phosphorelay proteins - $K, K_p, F, F_p, B, B_p, A, A_p$. There are two additional variables corresponding to the free levels of the phosphatases of Spo0A and Spo0F, denoted E and R, respectively. Finally, there are seven additional variables corresponding to the reaction intermediates involved in the phosphorylation, phosphotransfer and dephosphorylation reactions - $[K_pF], [F_pB], [B_pA], [KT], [KTF_p], [F_pR], [A_pE]$. Here, the variable T denotes ATP, which is assumed to be maintained at a constant value by the cell. Consequently, $[KT]$ is the KinA-ATP intermediate that mediates autophosphorylation and $[KTF_p]$ is the $[KT]$-Spo0F intermediate that mediates the dephosphorylation of Spo0F. These variables are not independent as they are constrained by the total concentrations of these proteins. There are six such constraints,

$$A_T = A + A_p + [B_pA] + [A_pE],$$

$$B_T = B + B_p + [B_pA] + [F_pB],$$

$$F_T = F + F_p + [F_pB] + [K_pF] + [KTF_p] + [F_pR],$$

$$K_T = K + K_p + [KTF_p] + [K_pF] + [KT],$$

$$R_T = [F_pR] + R,$$

$$E_T = [A_pE] + E.$$

Here, A_T, B_T, F_T, K_T, R_T and E_T denote the total concentrations of Spo0A, Spo0B, Spo0F, KinA, RapB, and Spo0E, respectively. Because of these six constraints, there are 11 free variables that describe the system, and are chosen to be $K, K_p, F, F_p, B, B_p, A, A_p, [K_pF], [F_pR], [A_pE]$. The remaining variables can be expressed as a combination of these variables and the total concentrations,

$$[B_pA] = A_T - A - A_p - [A_pE],$$

$$[F_pB] = B_T - B - B_p - [B_pA],$$

$$[KTF_p] = F_T - F - F_p - [F_pB] - [K_pF] - [F_pR],$$

$$[KT] = K_T - K - K_p - [KTF_p] - [K_pF],$$

$$R = R_T - [F_pR],$$

$$E = E_T - [A_pE].$$

The equations corresponding to these reactions are

$$\frac{dK}{dt} = -Tk_1K + k_{1p}[KT] + \nu_K[K_pF],$$

$$\frac{dK_p}{dt} = k_s[KT] - k_2K_pF + k_{2p}[K_pF],$$

$$\frac{dF}{dt} = -k_2K_pF + k_{2p}[K_pF] + \nu_P[KTF_p] + k_{5p}[F_pB] - k_5FB_p + \nu_R[F_pR],$$

$$\frac{dF_p}{dt} = \nu_K[K_pF] - k_3[KT]F_p + k_{3p}[KTF_p] - k_4F_pB + k_{4p}[F_pB] - k_8F_pR + k_{8p}[F_pR],$$

$$\frac{dB}{dt} = -k_4F_pB + k_{4p}[F_pB] + k_{7p}[B_pA] - k_7BA_p,$$

$$\frac{dB_p}{dt} = k_{5p}[F_pB] - k_5FB_p - k_6B_pA + k_{6p}[B_pA],$$

$$\frac{dA}{dt} = -k_6B_pA + k_{6p}[B_pA] + \nu_E[A_pE],$$

$$\frac{dA_p}{dt} = k_{7p}[B_pA] - k_7BA_p - k_9A_pE + k_{9p}[A_pE],$$

$$\frac{d[K_pF]}{dt} = k_2K_pF - (k_{2p} + \nu_K)[K_pF],$$

$$\frac{d[F_pR]}{dt} = k_8F_pR - (k_{8p} + \nu_R)[F_pR],$$

$$\frac{d[A_pE]}{dt} = k_9A_pE - (k_{9p} + \nu_E)[A_pE].$$

Here, the rate constants assigned to the different reactions as follows,

i. Autophosphorylation of K: k_1, k_{1p} are the association-dissociation rates for the reaction intermediate $[KT]$, and k_s is the rate of formation of K_p from this reaction intermediate.
ii. Phosphotransfer from K_p to F: k_2, k_{2p} are the association-dissociation rates for the reaction intermediate $[K_pF]$, and ν_K is the rate of formation of F_p from this reaction intermediate.
iii. Dephosphorylation of F_p by $[KT]$: k_3, k_{3p} are the association-dissociation rates for the reaction intermediate $[KTF_p]$, and ν_P is the rate of formation of F from this reaction intermediate.
iv. Phosphotransfer from F_p to B: k_4, k_{4p} are the association-dissociation rates for the reaction intermediate $[F_pB]$ from the reactants F_p and B.

v. Reverse phosphotransfer from B_p to F: k_5, k_{5p} are the association-dissociation rates for the reaction intermediate $[F_pB]$ from the reactants B_p and F.

vi. Phosphotransfer from B_p to A: k_6, k_{6p} are the association-dissociation rates for the reaction intermediate $[B_pA]$ from the reactants B_p and A.

vii. Reverse phosphotransfer from A_p to B: k_7, k_{7p} are the association-dissociation rates for the reaction intermediate $[B_pA]$ from the reactants A_p and B.

viii. Dephosphorylation of F_p by R: k_8, k_{8p} are the association-dissociation rates for the reaction intermediate $[F_pR]$, and ν_R is the rate of formation of F from this reaction intermediate.

ix. Dephosphorylation of A_p by E: k_9, k_{9p} are the association-dissociation rates for the reaction intermediate $[A_pE]$, and ν_E is the rate of formation of A from this reaction intermediate.

Parameter values are based on [1], $k_1 = k_2 = k_3 = \frac{1}{nM \cdot hr} = k_4 = k_5 = k_6 = k_7 = k_8 = k_9, k_{1p} = k_{2p} = k_{3p} = \nu_K = \nu_P = \frac{10^3}{hr} = k_{4p} = k_{5p} = k_{6p} = k_{7p} = k_{8p} = k_{9p} = \nu_R = \nu_E, k_s = \frac{10^2}{hr}, A_T = B_T = F_T = K_T = 1000nM, E_T = R_T = 100nM.$

The steady state for different total concentrations of Spo0F is computed by numerically integrating these equations using ode23s (Supp. Fig. S2H). For the computation with no reverse phosphotransfer, the corresponding rate constants are set to zero, $k_5 = k_7 = k_{4p} = k_{6p} = 0$. Similarly, for the computation with no Spo0F phosphatase, the corresponding parameters are set to zero, $R_T = k_8 = k_{8p} = \nu_R = k_3 = k_{3p} = \nu_P = 0$. There is an additional constraint as the reaction intermediate $[KTF_p]$ does not exist. Enforcing this constraint $([KTF_p] = 0)$ reduces the number of differential equations by one as the value of another reaction intermediate $[K_pF]$ is already determined, $[K_pF] = F_T - F - F_p - [F_pB] - [F_pR]$.

Supplementary Reference

1. Shinar G, Milo R, Martinez MR, Alon U (2007) Input output robustness in simple bacterial signaling systems. Proc Natl Acad Sci U S A 104(50): 19931-5.
