# Supplementary figures and images for: Dynamical Consequences of Bandpass Feedback Loops in a Bacterial Phosphorelay
Source: PLoS One. 2011 Sep 29;6(9):e25102. doi: 10.1371/journal.pone.0025102 (PMC3182994; doi:10.1371/journal.pone.0025102)

Figure S1

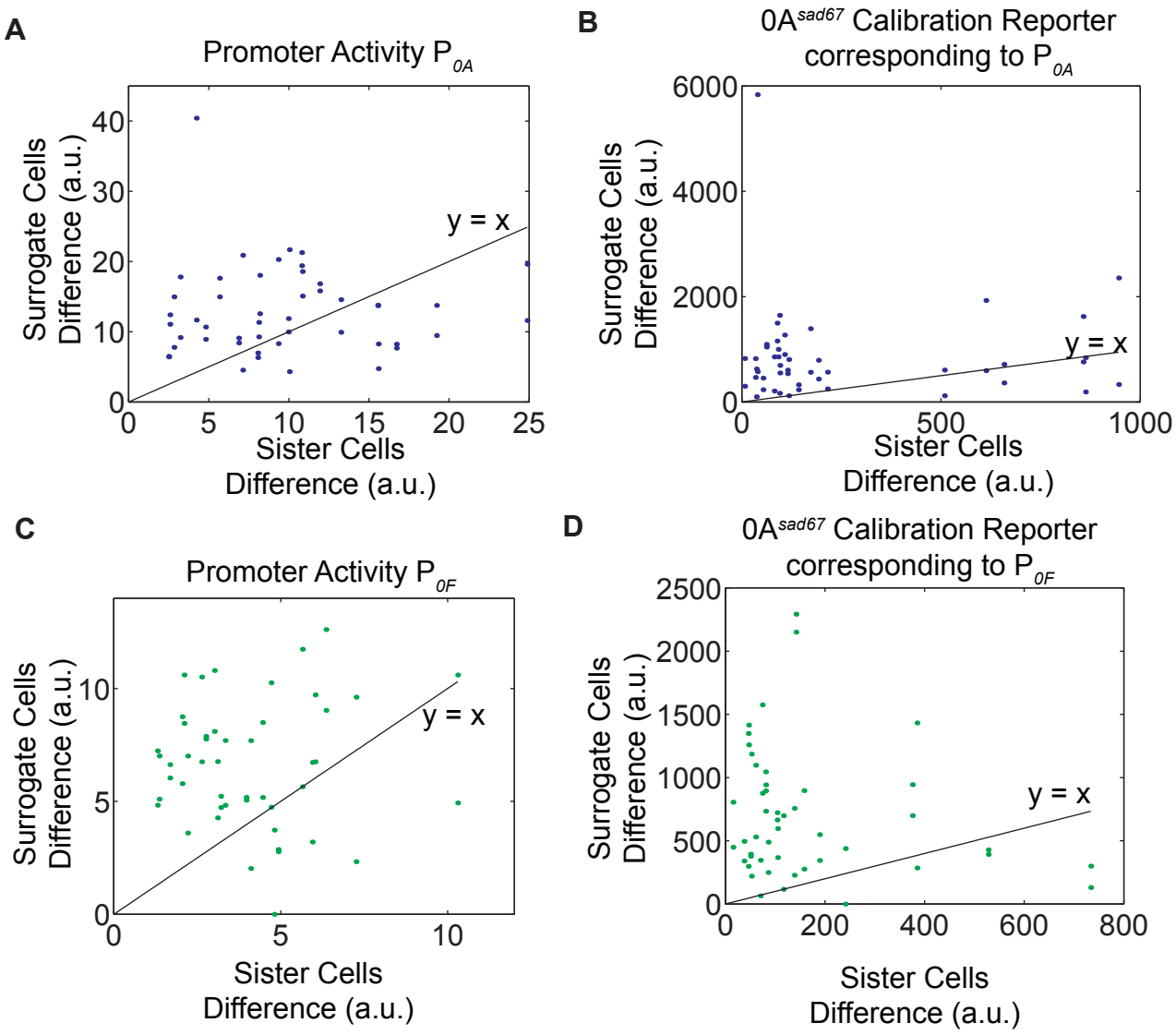

Supplement: Figure S1 — Variability between sister cells is typically smaller than between randomly chosen sister cell pairs. Difference between a cell and its sister and between the same cell and a randomly chosen surrogate sister cell is calculated for traces of P0A (A), Spo0Asad67 calibration reporter corresponding to the P0A bandpass measurement (B), P0F (C), and Spo0Asad67 calibration reporter corresponding to the P0F bandpass measurement (D). Each dot represents one cell, and has co-ordinates , where is the difference between sister cells and is the difference between surrogate cells. Difference metric for two given traces and is, , where is the minimum duration between the two traces and . In each case, most points lie above the straight line , indicating that the difference between sister cells is smaller than between surrogate sister cells. (PDF) [file pone.0025102.s002.pdf]

Figure S2

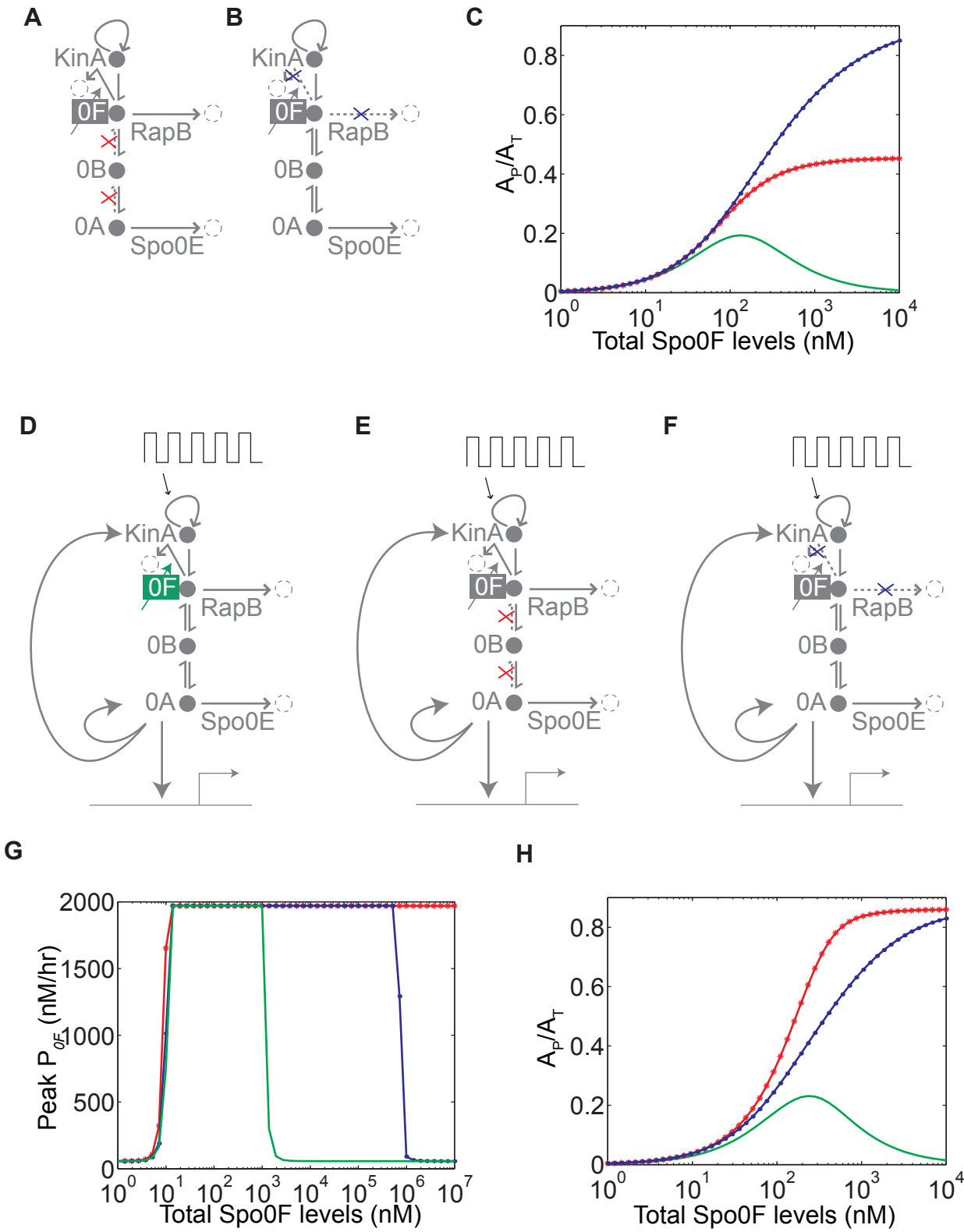

Supplement: Figure S2 — Post-translational bandpass in the model is due to drain of Spo0A phosphates via reverse phosphotransfer and Spo0F phosphatases. A–C. Diagrams of the core phosphorelay with no reverse phosphotransfer (A, red) and no Spo0F phosphatase (B, blue), for which the post-translational Spo0F bandpass computation is performed from the simple phosphorelay model (no pulsing or transcriptional feedbacks). Both curves are plotted in C, along with the curve from Fig. 2A (green). D–G. Diagrams of the phosphorelay circuit (D) and versions with no reverse phosphotransfer (E, red) and no Spo0F phosphatase (F, blue), for which the post-translational Spo0F bandpass computation is performed from the full model described in the main text (including pulsing and transcriptional feedbacks, Fig. 3). Peak P0F pulse amplitudes are plotted for different Spo0F induction levels (at steady state) for three cases: base parameters (G, green), no reverse phosphotransfer (G, red), and no Spo0F phosphatase (G, blue). The absence of Spo0F phosphatases shifts the repression threshold to significantly higher Spo0F values, representing drain of Spo0F phosphates by dilution due to cell growth. H. Green curve is the post-translational Spo0F bandpass computed from a more complicated version of the core phosphorelay (Text S1). The computation is repeated in the absence of reverse phosphotransfer (red) and Spo0F phosphatases (blue). (PDF) [file pone.0025102.s003.pdf]

Figure S3

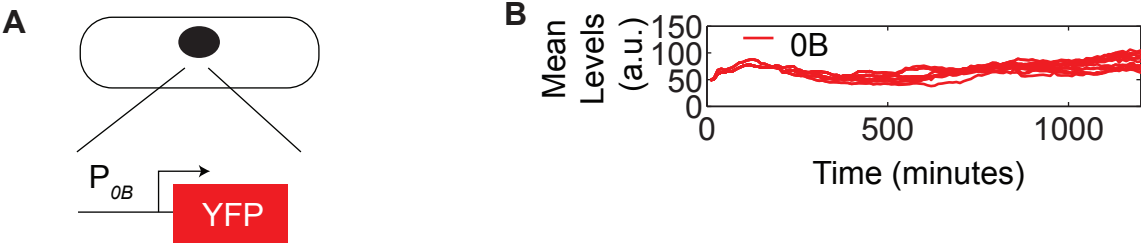

Supplement: Figure S3 — Mean fluorescence of a P 0B reporter changes less than twofold during sporulation initiation. A. Schematic of the strain with a YFP fluorescent reporter fused to P0B. The strain uses the plasmid ECE174 P0B-YFP (from lab stocks) integrated into an LS1 background. Imaging was done as described in the methods section, but on an Olympus IX-81 inverted microscope fitted with an ASI motorized stage, Hamamatsu Orca-ER camera, Sutter Lambda LS Xenon Arc lamp, and controlled using a combined Visual Basic-ImagePro software. B. Mean fluorescence levels are non-zero at the start and change less than twofold during sporulation initiation. (PDF) [file pone.0025102.s004.pdf]

Figure S4

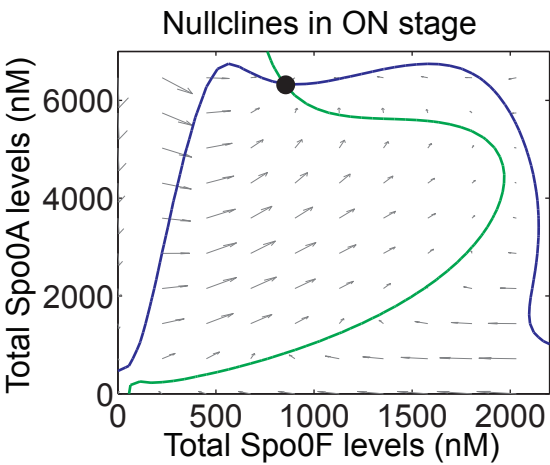

Supplement: Figure S4 — Qualitative dynamical picture under the P kinA = P 0A assumption is similar to the alternate P kinA = P 0F assumption. Nullclines in the “ON” stage in the case when , are similar to the case discussed in the main text with the assumption that , suggesting that this assumption is justified. These nullclines are plotted for same parameters as used in the main text with . (PDF) [file pone.0025102.s005.pdf]

# Figure S5

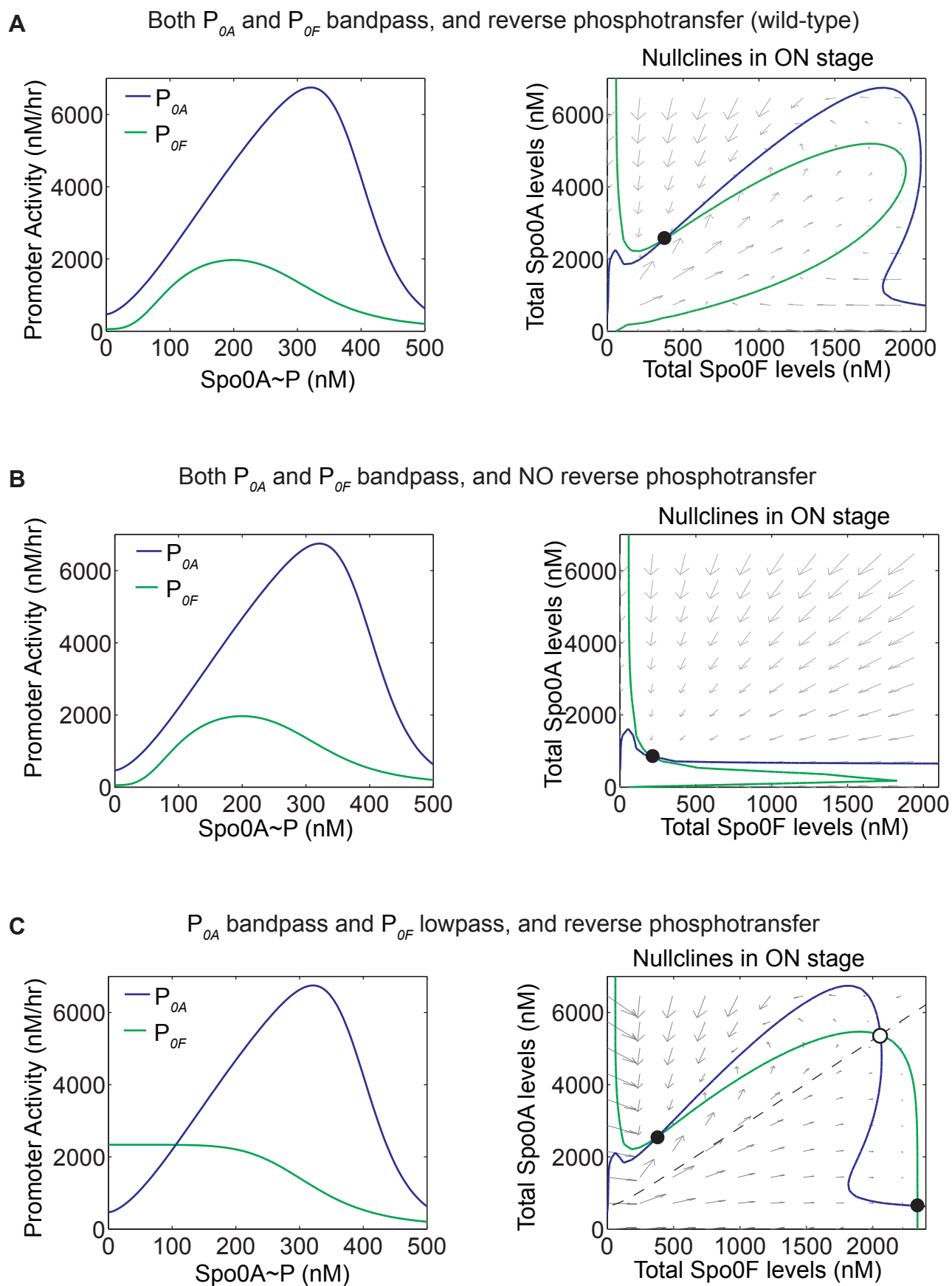

Figure S5

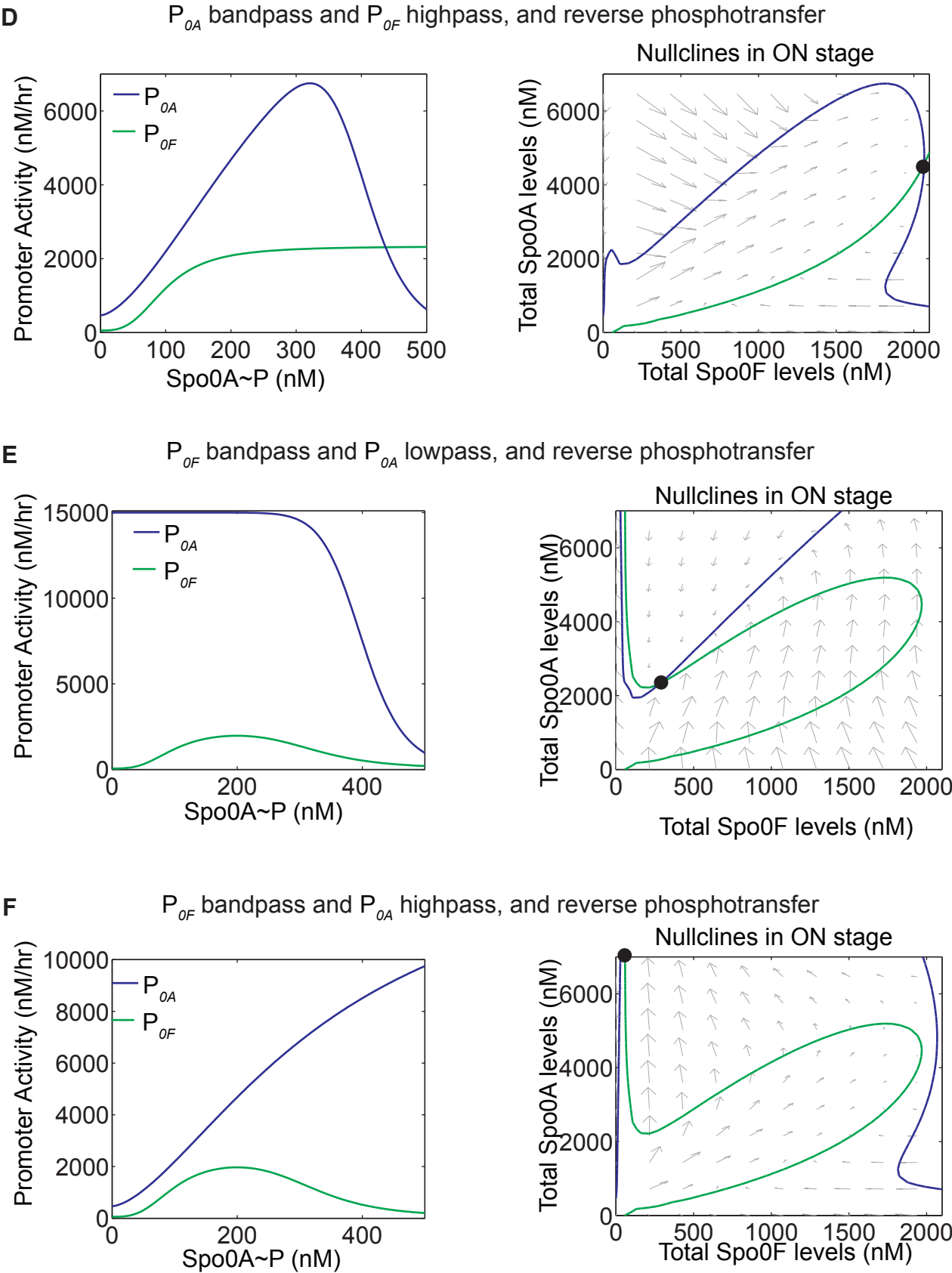

Supplement: Figure S5 — Effect of bandpass characteristics on the alternate state. The effect of bandpass characteristics on the alternate state are investigated by comparing “ON” phase planes in the following cases, A. Both P0A and P0F bandpass, and reverse phosphotransfer (wild-type case from Fig. 3C, considered in the main text) B. Both P0A and P0F bandpass, and NO reverse phosphotransfer: Transcriptional bandpasses remain the same as case A, but there is no post-translational bandpass. Nullclines orient differently from case A, so that the alternate state doesn't exist and its effect on dynamics is minimal. C. P0A bandpass, P0F lowpass (only repression), and reverse phosphotransfer: Nullclines are slightly perturbed from case A, with the appearance of a stable steady state, an unstable steady state (white circle), and the associated separatrix. The separatrix is computed by integrating equations backward in time starting from an initial condition near the unstable steady state. The additional stable steady state is similar in location to the alternate state. D. P0A bandpass, P0F highpass (only activation), and reverse phosphotransfer: Nullclines are slightly perturbed from case A, with the stable steady state situated like the alternate state. E. P0F bandpass, P0A lowpass (only repression), and reverse phosphotransfer: Nullclines are slightly perturbed from case A, but without significant change in the steady state location. F. P0F bandpass, P0A highpass (only activation), and reverse phosphotransfer: Nullclines are slightly perturbed from case A, but without significant change in the steady state location. In this case, the stable steady state is situated above the upper Y-axes limit. (PDF) [file pone.0025102.s006.pdf]
